# Global research trends and frontiers in ferroptosis in hepatocellular carcinoma: a bibliometric and visualization study

**DOI:** 10.3389/fonc.2024.1474496

**Published:** 2024-12-11

**Authors:** Lin Ning, Di Chen, Jie Han, Guanyue Xie, Jianguang Sun

**Affiliations:** ^1^ The First Clinical Medical College, Shandong University of Traditional Chinese Medicine, Jinan, China; ^2^ Affiliated Hospital of Shandong University of Traditional Chinese Medicine, Department of Hepatobiliary Medicine, Jinan, China

**Keywords:** ferroptosis, hepatocellular carcinoma, bibliometric analysis, research evaluation, research trends

## Abstract

**Background:**

Since the emergence of the hot topic of “ferroptosis,” numerous studies have explored its role in hepatocellular carcinoma (HCC), revealing its significance in the disease’s pathogenesis, progression, and treatment. However, there remains a significant gap in the quantitative analysis of ferroptosis in HCC. Therefore, this study aims to comprehensively assess the research progress and evolution in this field through bibliometric and citation analysis.

**Method:**

On June 27, 2024, the author conducted a literature search, extracting relevant publications from the Web of Science Core Collection (WOSCC) Science Citation Index Expanded (SCIE) spanning from January 2010 to December 2023. Subsequently, the compiled documents were subjected to bibliometric evaluation and analysis using visualization tools such as R package “bibliometrix”, CiteSpace and VOSviewer.

**Result:**

The search yielded 576 papers by 3,925 authors, encompassing contributions from 34 countries and 685 institutions, published across 250 journals, including 25,889 co-cited references from 2,600 journals. Notably, China leads with a significant publication count of 481 articles (accounting for 83.5%) and demonstrates the strongest collaboration with the United States. The multifaceted role of ferroptosis in hepatocellular carcinoma (HCC) has garnered considerable attention. In recent years, research into disease prognosis, the tumor microenvironment, and targeted therapies involving immunology has become key themes and emerging frontiers in this field.

**Conclusion:**

This study meticulously compiled and analyzed the current discourse and emerging perspectives on ferroptosis in HCC. Identifying research trends and hotspots offers valuable guidance for future investigations and provides a basis for the development of novel therapeutic strategies to improve HCC prognosis and treatment outcomes.

## 1 Introduction

Hepatocellular carcinoma (HCC) is the eighth most common cancer worldwide and the third leading cause of cancer-related deaths (1, 2). HCC accounts for 90% of all liver cancers, with a 5-year survival rate of less than 20% (3). As of 2020, it is estimated that there were 906,000 new cases and 830,000 deaths (4), with the incidence and mortality rates of HCC increasing annually worldwide, particularly in East Asia (5). Due to the high malignancy and poor prognosis of hepatocellular carcinoma (HCC), modern medical treatments for HCC, including surgical treatment and combined targeted immunotherapy, are diverse. However, the recurrence rate within five years after surgery remains as high as 70% (6). Therefore, understanding the pathogenesis of HCC and optimizing treatment strategies are particularly important in disease management.

Recent research has elucidated a significant link between ferroptosis—a regulated form of cell death characterized by iron-dependent lipid peroxidation—and various liver diseases (7), including nonalcoholic fatty liver disease, hepatic fibrosis, drug-induced liver injury, and liver tumors (8). Given its crucial role in cellular metabolism, redox homeostasis, and disease pathogenesis, most studies on ferroptosis have provided valuable insights into its dual nature in HCC progression (9, 10). Ferroptosis can facilitate tumor suppression by eliminating damaged cells and maintaining tissue health (11). Conversely, in established tumors, ferroptosis can be a double-edged sword, as tumor cells may develop resistance mechanisms to evade ferroptotic cell death, thus promoting survival under stress conditions such as oxidative stress or nutrient deprivation (12, 13). Additionally, impaired ferroptosis regulation, indicated by alterations in key regulators like GPX4 or SLC7A11, has been associated with spontaneous liver tumor development in experimental models (14–17). However, established tumors can exploit ferroptosis resistance pathways to progress, and evidence suggests that modulation of ferroptosis can influence HCC cell invasion and metastasis (18, 19). Despite the growing body of research on ferroptosis and its role in hepatocellular carcinoma (HCC), there remains a significant gap in the quantitative evaluation of research trends and collaborative networks within this domain. Bibliometric analysis, which employs statistical methods to systematically analyze large volumes of scientific literature, offers a unique opportunity to address this gap. By identifying key contributors, collaborative patterns, and emerging research themes, bibliometric studies provide valuable insights into the evolution of a research field (20–23). The lack of such quantitative analysis in ferroptosis and HCC research limits our ability to comprehensively understand global research dynamics, which could inform future studies and foster interdisciplinary collaboration. This study aims to fill this gap by providing a thorough bibliometric assessment of the existing literature on ferroptosis in HCC. The goal is to furnish clinicians and researchers with a synthesized overview of current developments and potential future trajectories for ferroptosis in HCC treatment and understanding.

## 2 Materials and methods

### 2.1 Literature search and screening

In this study, we utilized bibliometric methodologies to conduct a comprehensive analysis of the literature on ferroptosis and hepatocellular carcinoma (HCC). Our search was conducted through the Web of Science Core Collection (WoSCC) (https://www.webofscience.com/wos/woscc/basic-search), a well-established database recognized for its comprehensive coverage of high-quality scientific literature, including primary citation sources, peer-reviewed journals, and conference proceedings. Recent studies have highlighted the database’s expansion, particularly in the Science Citation Index Expanded (SCIE), which has improved coverage of various disciplines (24–26). On June 27, 2024, we retrieved articles published between January 2010 and December 2023 indexed within the SCIE of WoSCC.

The search strategy was carefully designed using the terms “hepatocellular carcinoma,” “primary liver cancer,” “primary liver carcinoma,” “HCC,” and “ferroptosis,” focusing on their occurrence in titles, abstracts, and keywords. We restricted the inclusion criteria to English-language, peer-reviewed articles and reviews to ensure the relevance and quality of the data. During the screening process, one non-English publication was excluded. Literature records were exported with the option “Full record and cited references” and formatted in plain text for further analysis.

Two independent reviewers (L.N. and D.C.) screened the initial dataset, systematically removing duplicates and irrelevant studies based on predefined inclusion criteria. In cases of disagreement, a third reviewer (J.H.) was consulted to resolve discrepancies. This process was guided by established bibliometric protocols to ensure the accuracy and reliability of the dataset.

### 2.2 Analytical methods and visualization

In this paper, we utilized various tools for bibliometric analysis and visualization. The annual publication count was organized and analyzed using Excel 2019. We employed VOSviewer (version 1.6.18) for country and institution analysis, journal and co-citation journal analysis, author and co-citation author analysis, and keyword co-occurrence analysis. VOSviewer is software that extracts key information from numerous publications and constructs collaboration networks, co-citation networks, and co-occurrence networks (27, 28). In the maps generated by VOSviewer, nodes represent items such as countries, institutions, journals, and authors. The size and color of the nodes indicate the quantity and classification of these items, respectively. The thickness of the lines between nodes reflects the degree of collaboration or co-citation among the items.

**Figure 1:**
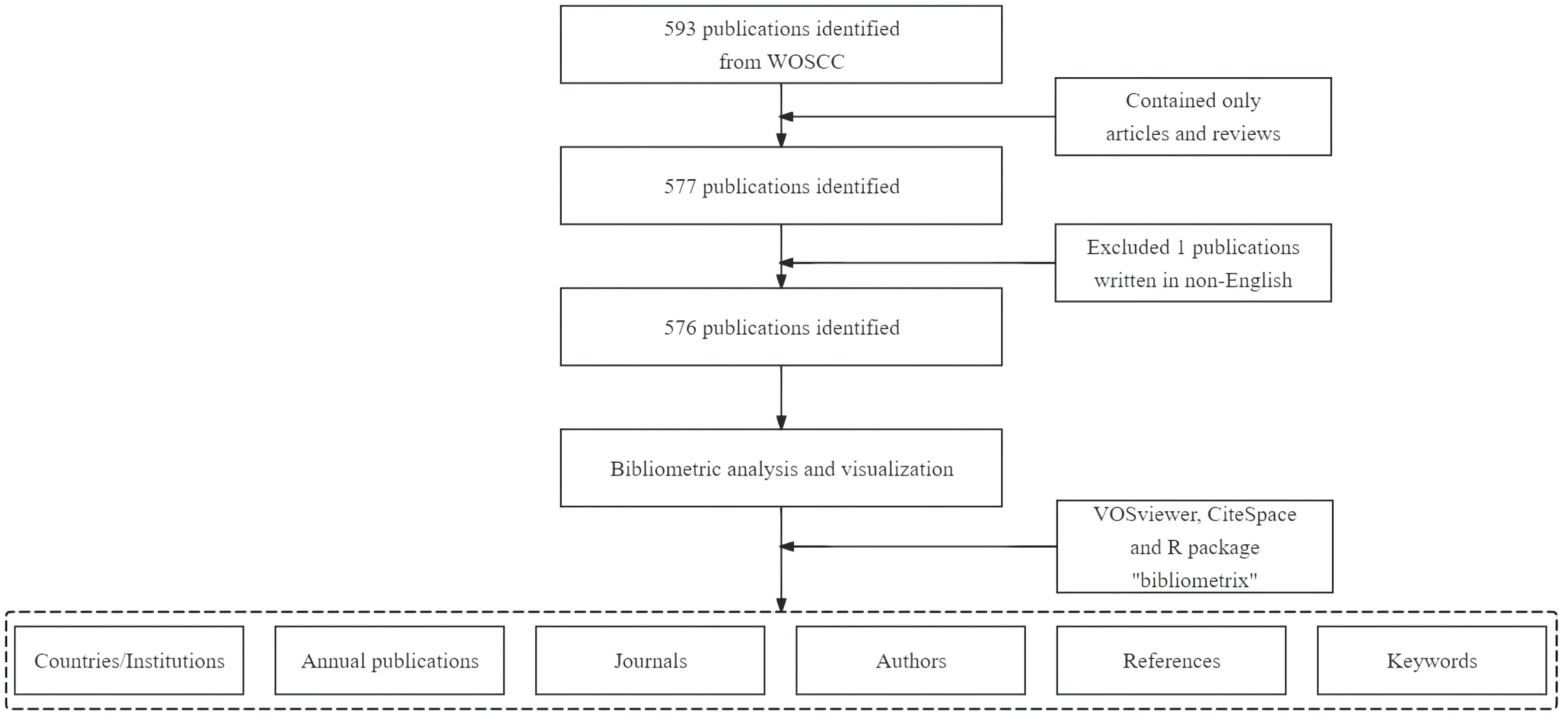
Flow chart of publication screening and analysis.

CiteSpace (version 6.1.R6) is another tool used for bibliometric analysis and visualization (29). In our study, CiteSpace was utilized to map the dual-map overlays of journals and to analyze references with citation bursts (30). We also used the R package “bibliometrix” (version 3.2.1) for thematic evolution analysis and to construct a global distribution network of publications on ferroptosis in the HCC field (31). The quartiles and impact factors of the journals were sourced from the 2020 edition of the Journal Citation Reports (32). The study’s flowchart is depicted in **Figure 1**.

## 3 Results

### 3.1 Analysis of publication trends

According to our search strategy, there were a total of 576 studies on ferroptosis and HCC (Hepatocellular Carcinoma) in the past decade, including 447 “articles” and 129 “reviews”.

**Figure 2:**
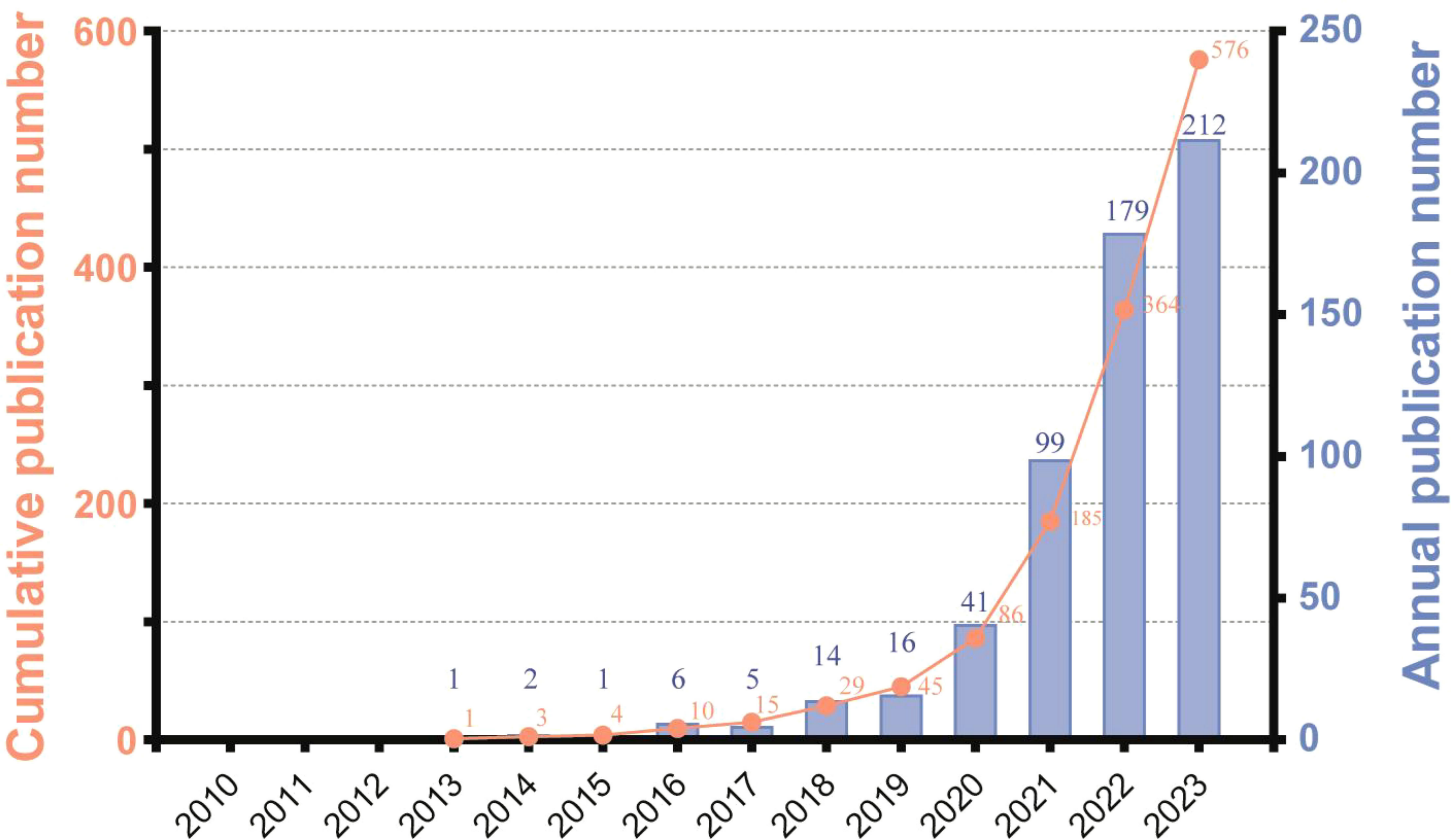
The annual publication quantity of ferroptosis in HCC.

The temporal analysis of publication trends reveals three distinct phases in ferroptosis and HCC research: Phase I (2013–2015), Phase II (2016–2018), and Phase III (2019–2023) (**Figure 2**). Phase I marks the foundational stage, with minimal publications focusing on the basic mechanisms of ferroptosis and its relevance to liver diseases. Phase II shows gradual growth, averaging 8.3 articles per year, reflecting initial explorations into ferroptosis-related biomarkers and its interplay with other cell death pathways. Phase III demonstrates rapid expansion, with an annual average exceeding 100 publications. This phase highlights the increasing focus on translational research, including ferroptosis-based therapies, immunotherapy, and combination treatments for HCC. The significant publication surge in Phase III, peaking at 212 in 2023, underscores the field’s growing clinical relevance. These phases illustrate the dynamic evolution of ferroptosis research, transitioning from foundational studies to cutting-edge applications, providing insights into future directions for HCC therapy.

It is noteworthy that the Web of Science Core Collection (WoSCC) underwent significant expansions in various directions at multiple points in time, including in 2005, 2015, and 2018. These expansions involved the inclusion of new types of literature, an increase in regional journals, and adaptations to open-access publishing models. The significant increase in the number of publications we retrieved can be partially attributed to these expansions in the Web of Science database (33).

### 3.2 Analysis of distribution of countries and institutions

These publications emanate from 34 distinct nations and 685 institutions. The nations with the highest publication outputs, constituting the top decile, are dispersed across Asia, North America, and Europe, with a pronounced emphasis on Asia (n=4) and Europe (n=5), as detailed in **Table 1**. Within this cohort, China is preeminent, contributing the majority of research articles (n=481, 83.5%), succeeded by the United States (n=57, 9.9%). Notable contributions are also observed from France, Japan, Germany, South Korea, and Italy, with a relatively equitable distribution of publications. China’s dominance in the research of ferroptosis in hepatocellular carcinoma (HCC) is unequivocal. The aggregate publications from China and the United States represent a substantial 93.4% of the total academic output in this domain.

**Table 1:** Top 10 countries and institutions in the research of ferroptosis in HCC.

Rank	Country	Counts	Institutions	Counts
1	People’s Republic of China	481(83.5%)	Sun Yat-sen University	29(5.0%)
2	United States	57(9.9%)	Central South University	27(4.7%)
3	France	15(2.6%)	Zhejiang University	23(4.0%)
4	Japan	15(2.6%)	Wenzhou Medical University	22(3.8%)
5	Germany	14(2.4%)	Guangzhou Medical University	19(3.3%)
6	South Korea	13(2.3%)	Shanghai Jiao Tong University	19(3.3%)
7	Italy	12(2.1%)	Fudan University	19(3.3%)
8	Singapore	5(0.9%)	Zhengzhou University	18(3.1%)
9	United Kingdom	5(0.9%)	Southern Medical University	16(2.8%)
10	Iran	4(0.7%)	Harbin Medical University	16(2.8%)

**Figure 3:**
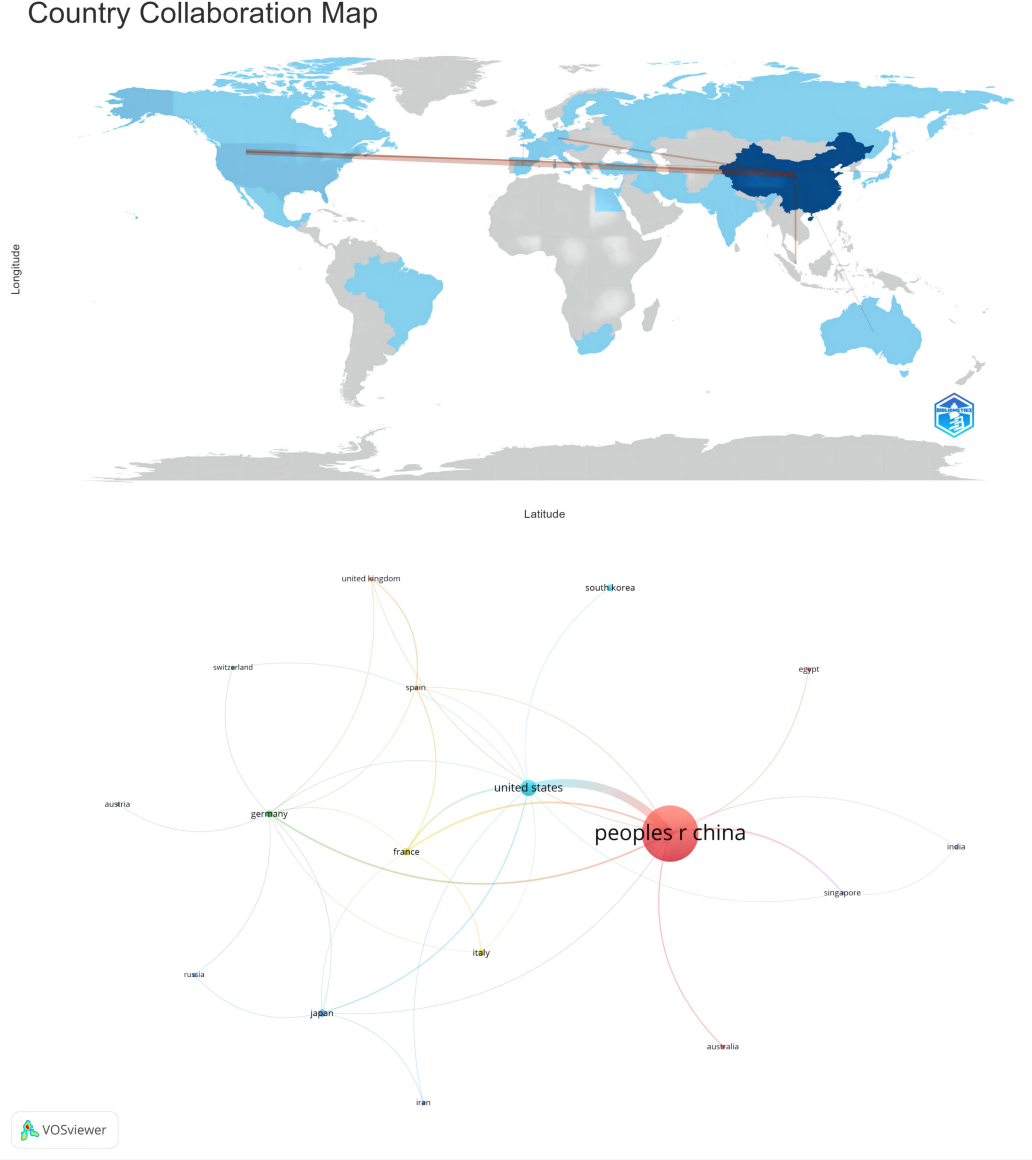
The geographical distribution and national visualization of ferroptosis research in HCC.

Subsequently, employing a filtration criterion of a minimum of two publications, we visualized and analyzed a collaborative network among the 34 nations, delineating the relationships and publication volumes of each, as depicted in **Figure 3**. **Figure 3** illustrates a robust tapestry of collaborative efforts among various nations. Particularly salient is the robust partnership between China and the United States. Additionally, China exhibits significant collaborative ties with France, Germany, and Japan. The United States, in turn, engages in proactive collaborative relationships with France, Japan, South Korea, and other nations. This collaborative framework underscores the global synergy in advancing the understanding of HCC and its associated pathological mechanisms.

**Figure 4:**
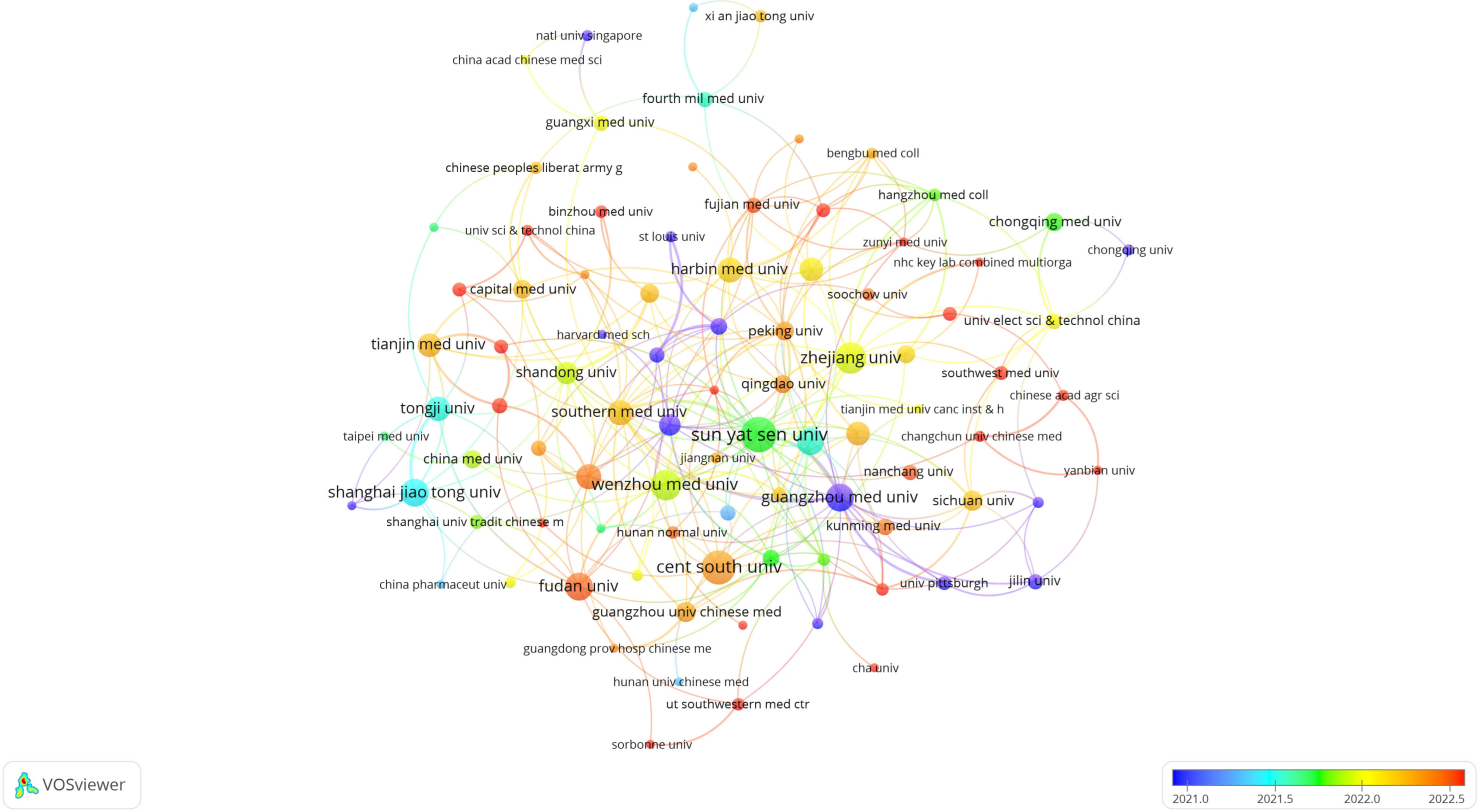
Visualization of research institutions on ferroptosis in HCC.

The top ten institutions by publication count are all from China. The most prolific institutions in publishing relevant scholarly articles are Sun Yat-sen University (n=29, 5.0%), Central South University (n=27, 4.7%), Zhejiang University (n=23, 4.0%), and Wenzhou Medical University (n=22, 3.8%). Following this, a visual representation was constructed of a collaboration network for 102 institutions that met or exceeded a threshold of three published papers, based on the volume and relational aspects of their scholarly contributions (**Figure 4**). Analysis of **Figure 4** reveals that Sun Yat-sen University enjoys a robust collaborative relationship with Southern Medical University and Guangzhou Medical University. Similarly, Central South University exhibits active collaboration with Guangzhou University of Chinese Medicine and Shanghai University of Traditional Chinese Medicine. A noteworthy partnership is also observed between Guangzhou University of Chinese Medicine and Southern Medical University. Additionally, Zhejiang University is closely connected with Wenzhou Medical University, which in turn has a strong collaborative tie with Fudan University, among other institutions. The network of these publishing institutions demonstrates a high degree of interconnectedness, reflecting the field’s capacity to garner the focused attention and investment from a multitude of institutions. This interconnectivity suggests a significant level of resource sharing and exchange, including research materials, data, and experimental apparatus, which may enhance collaborative research endeavors within the domain.

### 3.3 Analysis of active journals and co-cited journals

Publications related to ferroptosis in hepatocellular carcinoma (HCC) have appeared across 250 journals. **Table 2** lists the top 10 journals publishing research in this field. The journal with the highest number of publications is *Frontiers in Oncology* (n = 32, 5.6%), followed by *Frontiers in Cell and Developmental Biology* (n = 18, 3.1%), *Cell Death & Disease* (n = 15, 2.6%), and *International Journal of Molecular Sciences* (n = 15, 2.6%). Among these top 10 journals, all are classified in the top two Journal Impact Factor (JIF) quartiles (Q1 or Q2) according to the Journal Citation Reports (JCR). The journal with the highest impact factor is *Advanced Science* (IF = 14.3), with *Cell Death & Disease* being the next highest (IF = 8.0).

**Table 2:** Top 10 journals for the research of ferroptosis in HCC.

Rank	Journal	Counts	IF2023	JIF Quartile
1	Frontiers in Oncology	32(5.6%)	3.5	Q2
2	Frontiers in Cell and Developmental Biology	18(3.1%)	4.6	Q1
3	Cell Death & Disease	15(2.6%)	8.0	Q1
4	International Journal of Molecular Sciences	15(2.6%)	4.9	Q1
5	Frontiers in Pharmacology	13(2.3%)	4.4	Q1
6	Biomedicine & Pharmacotherapy	12(2.1%)	6.9	Q1
7	Frontiers in Immunology	11(1.9%)	5.7	Q1
8	Advanced Science	10(1.7%)	14.3	Q1
9	Frontiers in Genetics	10(1.7%)	2.8	Q2
10	Frontiers in Molecular Biosciences	9(1.6%))	3.9	Q2

**Figure 5:**
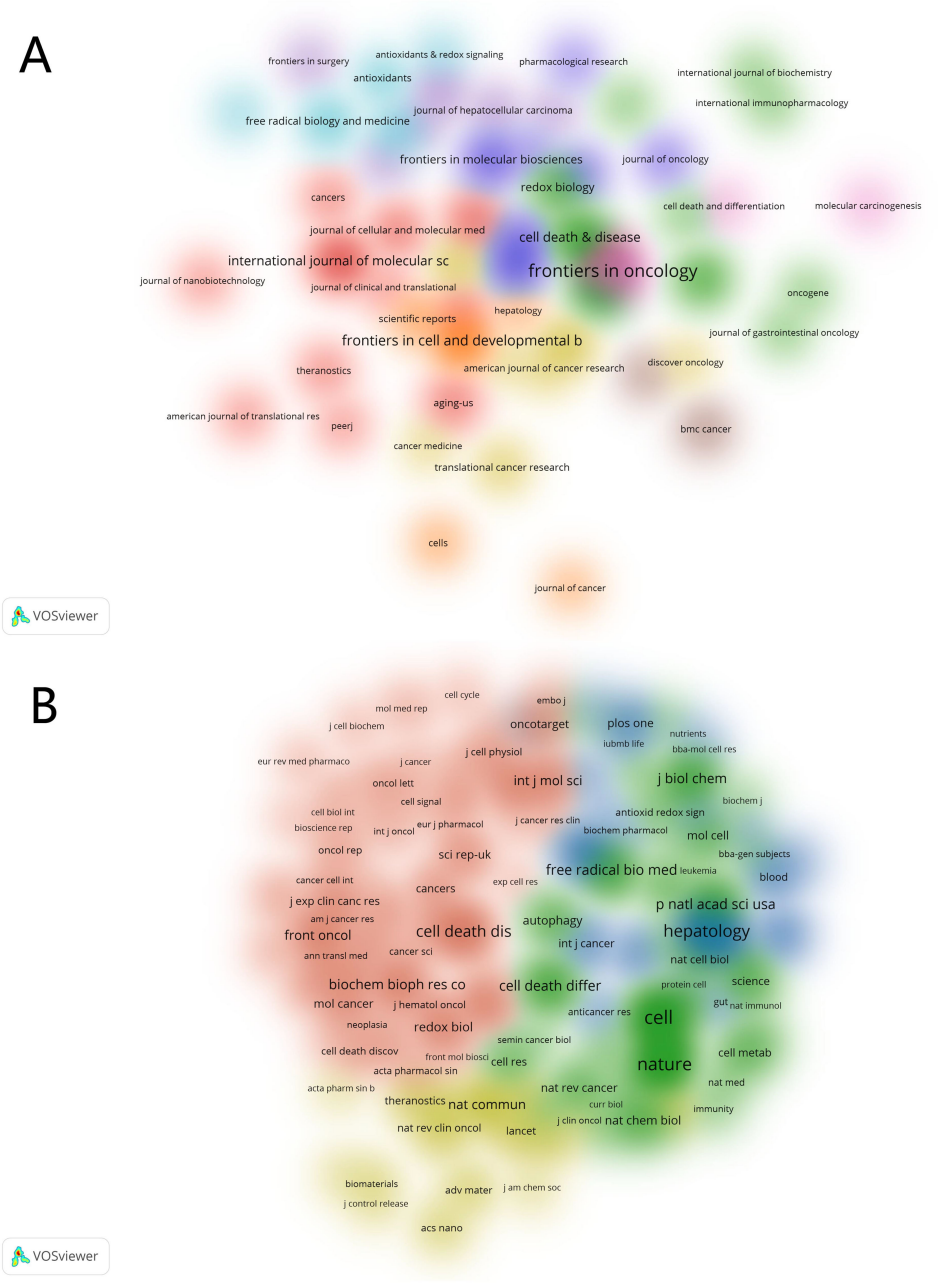
Visualization of the active and co-cited journals in the research of ferroptosis in HCC. **(A)** Active Journals. **(B)** Co-cited Journals.

Subsequently, we filtered out 58 journals based on a minimum publication threshold of three and created two visualizations: the distribution of active journals (**Figure 5A**) and the co-citation network of journals (**Figure 5B**). **Figure 5A** represents a density map of journals actively publishing on ferroptosis and HCC, where the size of each label corresponds to the number of publications, and the proximity of journals indicates the degree of thematic similarity. Prominent journals, such as *Frontiers in Oncology* and *Cell Death & Disease*, appear at the center, reflecting their significant contributions to the field. **Figure 5B** depicts the co-citation network of journals, highlighting the relationships between journals frequently cited together. The size of the nodes represents the frequency of co-citations, while the clustering (indicated by different colors) reveals distinct research domains or subfields within ferroptosis and HCC. For instance, clusters associated with *Hepatology* and *Nature* are indicative of foundational and high-impact research. These visualizations provide an overview of the academic landscape and key contributors, offering insights into the interconnectedness and influence of various journals in this research area.

We have also listed the top 10 journals by citation count, as detailed in **Table 3**. The high citation frequency of journals such as *Cell, Nature*, and *Hepatology* indicates a significant level of trust and reliance on the findings published in these prestigious periodicals within the field. These analyses indicate that research on ferroptosis in hepatocellular carcinoma (HCC) is predominantly published in specialized journals within the fields of oncology, cell and developmental biology, as well as cell death and disease. Furthermore, they enable us to discern which journals in specific domains pay closer attention to the subject of ferroptosis in HCC, thereby reflecting the current trends and the focal points of research interest.

**Table 3:** Top 10 co-cited journals for the research of ferroptosis in HCC.

Rank	Co-cited Journal	Co-citation	IF2023	JIF Quartile
1	Cell	1195	45.5	Q1
2	Nature	1076	69.5	Q1
3	Hepatology	920	12.4	Q1
4	Cell Death & Disease	783	8.0	Q1
5	Cell Death & Differentiation	608	13.7	Q1
6	Proceedings of the National Academy of Sciences of the United States of America	570	12.7	Q1
7	Free Radical Biology and Medicine	569	8.1	Q1
8	Cancer Research	565	13.7	Q1
9	The Journal of Biological Chemistry	556	5.5	Q2
10	Biochemical and Biophysical Research Communications	538	3.5	Q3

### 3.4 Analysis of authors and co-cited authors

**Figure 6:**
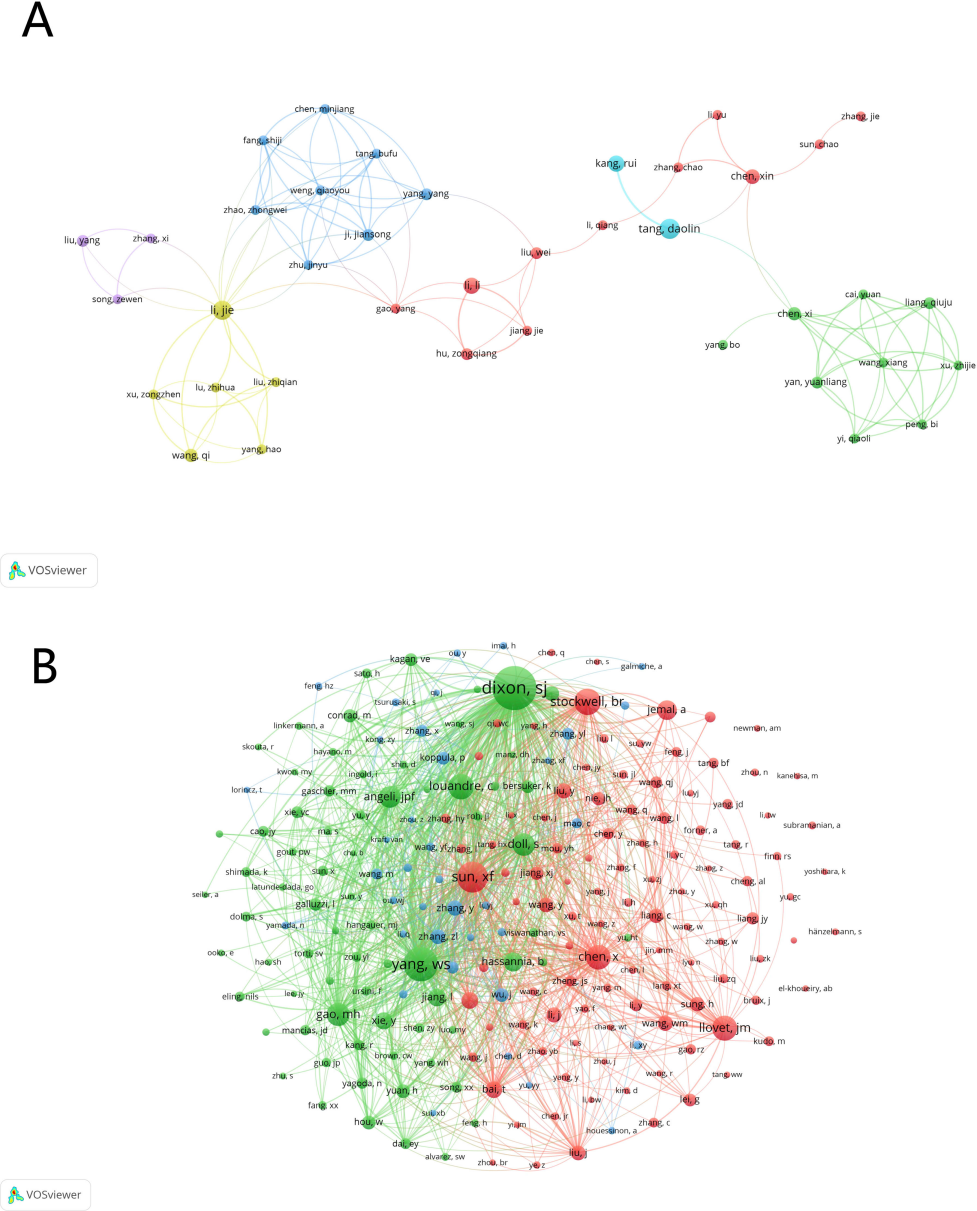
The visualization of active authors and co-cited authors of ferroptosis in HCC research. **(A)** Active authors. **(B)** Co-cited authors.

To ensure the accuracy of the author analysis and to minimize the occurrence of author name duplication, we combined the information of the authors’ affiliated institutions in our analysis to distinguish whether authors with the same name are the same person. Upon analysis, a total of 3,925 researchers have made contributions in the field of ferroptosis and HCC. By setting a threshold of at least 3 published papers, we constructed an author collaboration network (**Figure 6A**). Among these authors, the top five contributors, all from China, are Tang Daolin, Li Jie, Kang Rui, Li Li, and Wang Yi, with 11, 9, 7, 7, and 7 publications respectively. **Figure 6A** illustrates the frequent collaborative interactions among these authors, particularly highlighting the close collaborations between Tang Daolin and Kang Rui; Li Li and Hu Zongqiang; and Li Jie and Wang Qi, who also have significant publication volumes.

For the co-cited author network (**Figure 6B**), we set a minimum citation threshold of 20. Among the 3,925 authors, seven have been cited more than 200 times, as detailed in **Table 4**. The most frequently co-cited author is Dixon S.J., with 550 citations, followed by Yang W.S. (n=362) and Sun X.F. (n=297). **Figure 6B** clearly shows that these highly co-cited authors also have close collaborative relationships.

**Table 4:** Top 10 authors and co-cited authors on research of ferroptosis in HCC.

Rank	Authors	Counts	Co-cited Authors	Citations
1	Tang, Daolin	10	Dixon, S.J.	550
2	Li, Jie	9	Yang, W.S.	362
3	Kang, Rui	7	Sun, X.F.	297
4	Li, Li	7	Stockwell, B.R.	230
5	Wang, Yi	7	Louandre, C.	219
6	Chen, Gang	6	Llovet, J.M.	209
7	Chen, Xin	6	Chen, X.	206
8	Wang, Hao	6	Gao, M.H.	184
9	Chauffert, Bruno	5	Doll, S.	176
10	Chen, Xi	5	Angeli, J.P.F.	162

These analyses demonstrate the strong presence and contributions of Chinese researchers in the field of HCC and ferroptosis. The collaborative relationships and publication volumes indicate the existence of core research teams in this field. Furthermore, the co-citation network reveals that these highly cited authors are not only influential but also likely part of a tightly connected research community.

### 3.5 Analysis of co-cited references and citation bursts

**Figure 7:**
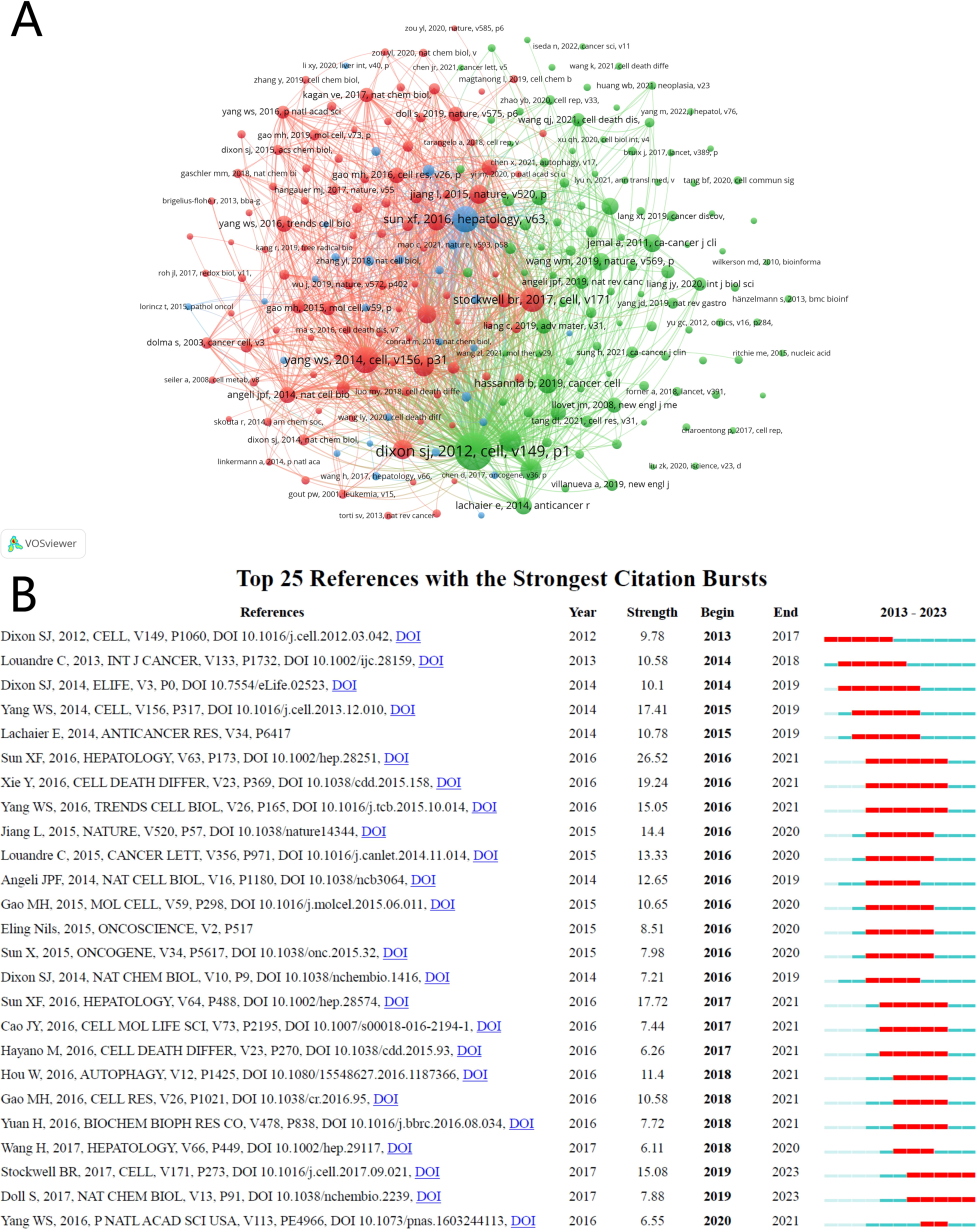
The visualization of co-cited references of ferroptosis in HCC research. **(A)** Co-cited references. **(B)** Top 25 references with strong citation bursts.

Between 2010 and 2023, research on HCC and ferroptosis amassed a total of 25,889 academic citations. We constructed a co-citation network graph (**Figure 7A**) using literature cited at least 15 times. The top three cited publications originated from the United States and China, published in “Cell” and “Hepatology,” respectively (34–36). Detailed information on the top ten cited papers is provided in **Table 5**, **Figure 7A** illustrates significant co-citation relationships among these publications.

**Table 5:** Top 10 co-cited references on research of ferroptosis in HCC.

Rank	Co-cited reference	Citations
1	Ferroptosis: an iron-dependent form of nonapoptotic cell death. Cell. 2012 May 25;149(5):1060-72. doi: 10.1016/j.cell.2012.03.042.	328
2	Activation of the p62-Keap1-NRF2 pathway protects against ferroptosis in hepatocellular carcinoma cells. Hepatology. 2016 Jan;63(1):173-84. doi: 10.1002/hep.28251.	175
3	Regulation of ferroptotic cancer cell death by GPX4. Cell. 2014 Jan 16;156(1-2):317-331. doi: 10.1016/j.cell.2013.12.010.	173
4	Ferroptosis: A Regulated Cell Death Nexus Linking Metabolism, Redox Biology, and Disease. Cell. 2017 Oct 5;171(2):273-285. doi: 10.1016/j.cell.2017.09.021.	157
5	Iron-dependent cell death of hepatocellular carcinoma cells exposed to sorafenib. Int J Cancer. 2013 Oct 1;133(7):1732-42. doi: 10.1002/ijc.28159.	125
6	Metallothionein-1G facilitates sorafenib resistance through inhibition of ferroptosis. Hepatology. 2016 Aug;64(2):488-500. doi: 10.1002/hep.28574.	122
7	Pharmacological inhibition of cystine-glutamate exchange induces endoplasmic reticulum stress and ferroptosis. Elife. 2014 May 20;3:e02523. doi: 10.7554/eLife.02523.	117
8	Ferroptosis: process and function. Cell Death Differ. 2016 Mar;23(3):369-79. doi: 10.1038/cdd.2015.158.	105
9	Targeting Ferroptosis to Iron Out Cancer. Cancer Cell. 2019 Jun 10;35(6):830-849. doi: 10.1016/j.ccell.2019.04.002.	104
10	Ferroptosis as a p53-mediated activity during tumor suppression. Nature. 2015 Apr 2;520(7545):57-62. doi: 10.1038/nature14344.	102

The term “sudden surge in citations” describes a phenomenon where a particular research field or direction experiences a rapid increase in the number of citations for specific areas or papers within a short period. This occurrence often indicates that new research findings or breakthroughs have emerged in the field, leading to a surge in attention and citations. We have listed the top 25 publications in **Figure 7B** that exhibit a surge in citation counts and a strong citation burst. Each bar in the graph represents a year, with red bars indicating intense citation bursts. The first notable surge occurred in 2013, with the most recent in 2020. The publication with the highest citation burst intensity was Sun XF et al.’s 2016 article in “Hepatology,” titled “Activation of the p62-Keap1-NRF2 pathway protects against ferroptosis in hepatocellular carcinoma cells,” with a burst period spanning 2016-2021. On average, these 25 publications experienced a citation burst intensity of 12 times, lasting between 2 to 5 years.

### 3.6 Analysis of keywords and trend topics

**Figure 8:**
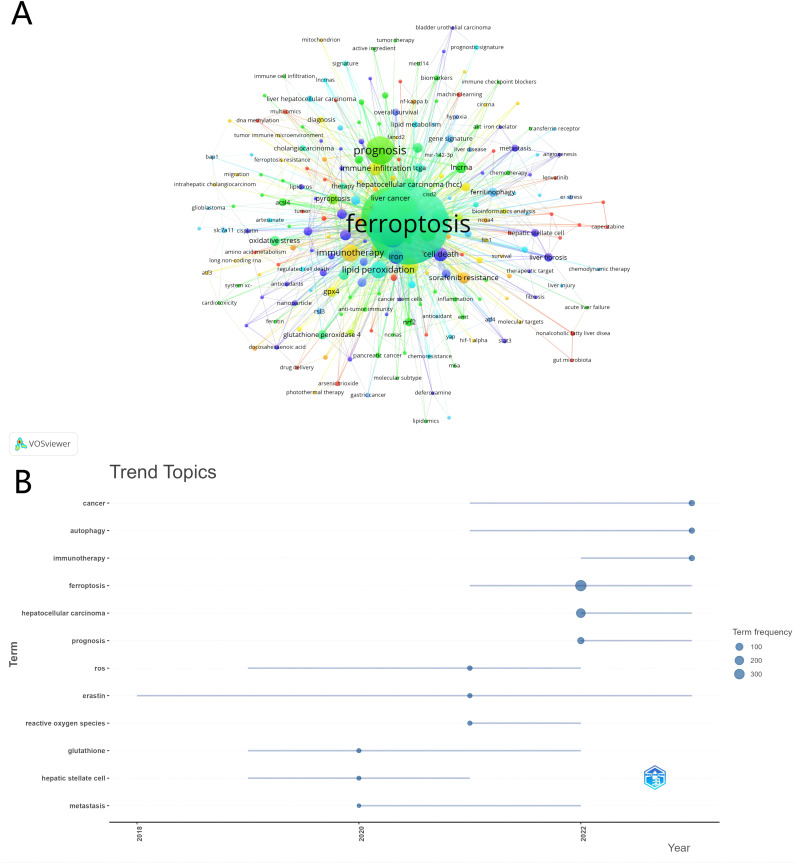
Keywords and trend topics. **(A)** Keyword cluster analysis. **(B)** trend topic analysis.

We conducted an in-depth exploration of popular topics within a specific research field through keyword co-occurrence analysis. In our analysis of 576 papers, we identified 2,035 keywords. To ensure the rigor of our analysis, we filtered out keywords that appeared fewer than three times. **Figure 8A** displays the high-frequency keywords in the “ferroptosis and HCC” domain, with the thickness of the lines between nodes indicating the strength of the associations between keywords. **Table 6** lists the top 30 keywords ranked by frequency. In addition to “ferroptosis” and “hepatocellular carcinoma,” keywords such as “prognosis,” “sorafenib,” “autophagy,” “tumor microenvironment,” “lipid peroxidation,” and “apoptosis” stand out with high node connectivity.

**Table 6:** Top 30 keywords on research of ferroptosis in HCC.

Rank	Keywords	Counts	Rank	Keywords	Counts
1	ferroptosis	377	16	iron metabolism	15
2	hepatocellular carcinoma	216	17	liver cancer	13
3	prognosis	62	18	lncrna	13
4	sorafenib	44	19	sorafenib resistance	13
5	cancer	32	20	pyroptosis	12
6	autophagy	29	21	ros	12
7	immunotherapy	27	22	oxidative stress	11
8	lipid peroxidation	26	23	erastin	10
9	tumor microenvironment	26	24	gpx4	10
10	hcc	25	25	immune	10
11	apoptosis	24	26	necroptosis	10
12	cell death	18	27	biomarker	9
13	immune infiltration	18	28	ferritinophagy	9
14	iron	18	29	glutathione peroxidase 4	9
15	hepatocellular carcinoma (hcc)	15	30	reactive oxygen species	9

Further analysis of the trend in research topics (**Figure 8B**) reveals the evolution of the research focus. In relatively early studies, the appearance of “glutathione” and “hepatic stellate cell” indicates an increased interest in liver diseases, particularly in cell types and antioxidant mechanisms related to liver fibrosis and cirrhosis. Additionally, “metastasis” as a critical topic in cancer research shows a sustained interest in the mechanisms of cancer spread. Over time, keywords related to oxidative stress, such as “ferroptosis” and “reactive oxygen species,” have gained more attention, possibly reflecting a new interest in the role of oxidative stress in disease development. In recent years, research has primarily focused on cancer treatment and mechanisms, with keywords like “autophagy” and “immunotherapy” frequently appearing in the context of cancer treatment. Moreover, “hepatocellular carcinoma” as a specific type of cancer has received significant attention. From the term frequency analysis, “prognosis” has also emerged as a central topic in the research.

Overall, the frequency and trend of these keywords reveal an in-depth exploration of disease mechanisms, treatment strategies, and specific pathological processes in the field of medical research, as well as a shift in research focus.

## 4 Discussion

Ferroptosis is a regulated form of cell death characterized by iron-dependent lipid peroxidation, which plays a significant role in hepatocellular carcinoma (HCC). It is associated with the pathogenesis, progression, and response to therapy of the disease, making it a potential therapeutic target for controlling HCC (37, 38).

Ferroptosis serves multifaceted roles in HCC. Initially, by inducing oxidative stress and lipid peroxidation in cancer cells, ferroptosis can inhibit their proliferation and growth, thereby slowing the progression of HCC (39). Secondly, ferroptosis is related to the tumor microenvironment and immune response, enhancing the anti-tumor immunity (40). Furthermore, ferroptosis can improve the efficacy of certain chemotherapeutic drugs and targeted therapies by affecting the sensitivity of liver cancer cells to these treatments (41). Therefore, investigating the mechanisms of ferroptosis in HCC is expected to provide new insights and strategies for the treatment of HCC.

This study analyzes publication trends, geographic distribution, collaborative networks, and research hotspots to enhance our understanding of the role of ferroptosis in HCC and to spur the development of innovative treatment strategies for this malignancy.

### 4.1 Countries/institutions and their cooperation

Our research findings indicate that over the past decade, there has been a significant increase in studies on ferroptosis in HCC, with a marked surge from 2019 to 2021, and peaking in 2023. This upward trend underscores the growing scientific interest in the correlation between ferroptosis and HCC. Publications from the top ten countries span Asia, North America, and Europe. China stands out, contributing the majority of research articles (83.5%), followed by the United States (9.9%). The combined output from China and the United States accounts for 93.4% of the total academic contributions in this field. The dominance of China in ferroptosis research in HCC is evident and aligns with previous findings.

While China and the United States exhibit strong research collaboration, their engagement with other global partners appears relatively limited. China has collaborated with over ten countries, occupying a central position in the collaboration network. However, there is potential for further strengthening alliances with European and American countries. This collaborative framework highlights the global synergistic efforts in enhancing the understanding of HCC and its associated pathological mechanisms.

Regarding the institutions associated with these publications, Chinese institutions prominently occupy the top ten, reflecting the crucial role of Chinese researchers in this field. This phenomenon may be attributed to the higher prevalence of HCC in Asia and the increased emphasis on cancer research in developing countries (42, 43). Sun Yat-sen University emerges as the most prolific, maintaining extensive collaborations with institutions like Southern Medical University, Central South University, and Zhejiang University, likely facilitated by geographic proximity and shared research interests.

Overall, China’s dominant role in ferroptosis and HCC research can be attributed to several interconnected factors. First, substantial government investment in scientific research has provided a robust infrastructure, facilitating cutting-edge studies in oncology and related fields. According to recent reports, China allocates significant funding to cancer research, supporting both basic and translational studies (44). This financial commitment is further bolstered by policies promoting high-impact publications, incentivizing researchers to contribute actively to international journals. Second, as mentioned earlier, the high incidence of HCC in China has been pivotal in shaping the research landscape. As one of the countries with the highest burden of liver cancer globally, there is an urgent need to explore innovative therapeutic strategies, including ferroptosis-targeted treatments. This epidemiological reality drives research priorities and fosters interdisciplinary collaborations (45). Third, China’s strong international collaboration networks have enhanced the output and quality of its research. As our analysis highlights, partnerships between China and the United States, as well as collaborations with European countries like Germany and France, underscore the synergistic efforts in advancing HCC understanding. These collaborations bring diverse expertise and resources, strengthening the impact of Chinese research. Finally, the increasing focus on bibliometric analysis and visualization techniques has enabled Chinese researchers to efficiently identify emerging trends and research frontiers, aligning their work with global priorities. Together, these factors have positioned China as a leader in publication volume in this field.

### 4.2 Journals and citation circumstances

As shown in **Table 2**, the top ten journals publishing research on HCC and ferroptosis are committed to quality and impact, all classified in the top two JIF quartiles (Q1 or Q2) according to the Journal Citation Reports (JCR). This esteemed group includes “*Frontiers in Oncology*,” which has published 32 papers on the topic, and “*Advanced Science*,” which has the highest impact factor. The prominence of these journals among the top ten highlights the importance of oncology, cell and developmental biology, and cell death and disease as key research areas within this field. According to a study published in *Scientometrics* in 2016 (46), over 25% of SCIE articles were published in top-quartile (Q1) journals, highlighting their central role in the academic community. Due to their high impact and widespread recognition, Q1 journals attract a substantial volume of high-quality research, driving academic dissemination while reflecting the concentration of premium research resources in leading institutions. Furthermore, the current research evaluation system’s bias toward Q1 journals enhances their appeal, creating a positive feedback loop between submission preferences and academic recognition. However, this dynamic also results in greater opportunities for research in trending fields to appear in Q1 journals, while studies in less prominent areas face fewer opportunities for publication. With the rise of open-access publishing, some high-impact open-access journals have entered the Q1 category, further increasing the proportion of articles published in these journals. Therefore, while Q1 journals dominate the dissemination of academic findings, over-reliance on Q1 metrics in evaluation systems risks exacerbating the unequal distribution of research resources and opportunities. A more balanced evaluation approach is needed to support the diversification of academic research.

High citation counts indicate that influential studies have made significant contributions to advancing knowledge in this specific domain. In the field of ferroptosis and HCC, citation analysis reveals a concentration of influential research. The most cited publication is “*Ferroptosis: an iron-dependent form of nonapoptotic cell death*” by Dixon S.J., published in “*Cell*.” This pioneering paper introduces the discovery and characteristics of ferroptosis, providing potential targets for developing therapeutic strategies against specific types of tumors, and has become a milestone in the research area. The second most cited paper, authored by Sun X.F. from China and published in “*Hepatology*,” is titled “*Activation of the p62-Keap1-NRF2 pathway protects against ferroptosis in hepatocellular carcinoma cells.*” This study reveals that the activation of the p62-Keap1-NRF2 pathway plays a crucial role in protecting against ferroptosis in HCC, leading scholars to explore key pathways involved in ferroptosis and shifting the research focus significantly. The emergence of these studies has laid the foundation for subsequent research on key molecules such as glutathione peroxidase 4 (GPX4) (47), system Xc- (SLC7A11) (48), and critical pathways like the Nrf2-ARE (49) and p53 (50) pathways. Notably, while China has the highest output in this field, the most cited publications predominantly come from the United States and Europe. This discrepancy indicates that although Chinese scholars have made outstanding contributions to this domain, the quality of their publications still needs improvement to achieve broader global impact.

### 4.3 Research hotspots and frontiers

The analysis of the “Citation Bursts” section reveals pivotal moments within the fields of HCC and ferroptosis that have garnered academic attention (51). Starting from 2013 and particularly by 2020, a significant increase in citation numbers reflects substantial research advancements in this domain. A publication by Sun X.F. et al. in the journal *Hepatology* in 2016, which discusses the protective role of the p62-Keap1-NRF2 pathway in modulating the susceptibility of HCC cells to ferroptosis, stands out with the highest intensity of citation bursts. The impact of this particular study was evident during the burst period from 2016 to 2021, highlighting the potential role of NRF2 in the therapeutic response of HCC cells to ferroptosis-targeted treatments (18, 35, 52, 53).

Delving into the “Keywords and Trend Topics” analysis unveils the evolution of research focus areas. High-frequency keywords identified through co-occurrence analysis reflect the core themes and focal areas within the field. “Ferroptosis” and “hepatocellular carcinoma” are central to the research discourse, while other keywords such as “prognosis,” “sorafenib,” “autophagy,” (54, 55) “tumor microenvironment,” (56) “lipid peroxidation,” and “apoptosis” exhibit high node connectivity (57). These keywords not only indicate the multifaceted nature of the field but also underline its translational significance. For instance, the increased focus on “autophagy” and “immunotherapy” (58, 59) suggests potential therapeutic avenues that integrate ferroptosis modulation to improve HCC treatment outcomes. Similarly, the emphasis on “tumor microenvironment” points to opportunities for combining ferroptosis-based therapies with strategies targeting immune infiltration to enhance anti-tumor responses.

Recent trends further reveal that identifying novel biomarkers for early detection and prognosis has become a key focus, with applications in precision medicine. Innovative therapies, including ferroptosis-inducing agents combined with immunotherapeutic approaches, are actively being explored for their potential to improve HCC prognosis and survival rates (60–62). Researchers are increasingly employing systems biology approaches (63, 64), high-throughput screening (19, 65), and computational models (66, 67), making future research more interdisciplinary and clinically actionable.

In summary, the citation bursts and keyword trends highlight the dynamism and translational potential of research in HCC and ferroptosis. These analyses not only reflect the current state of knowledge but also signal future directions, particularly in developing ferroptosis-targeted therapies to improve clinical outcomes. The discussions underscore the vibrancy of the field and its potential to advance patient care, offering new hope for individuals afflicted with this devastating disease.

Ferroptosis has emerged as a pivotal mechanism in HCC, offering novel therapeutic opportunities. Despite significant advancements, several key areas require further exploration to fully harness ferroptosis for clinical applications. One promising direction is the integration of ferroptosis-targeted therapies with existing immunotherapy and chemotherapy regimens. Ferroptosis has been shown to modulate immune responses by altering the tumor microenvironment, particularly through the regulation of immune cell infiltration and oxidative stress mechanisms (7, 68). Combining ferroptosis-inducing agents with immune checkpoint inhibitors or traditional chemotherapeutics could potentially overcome treatment resistance and enhance therapeutic efficacy. Furthermore, understanding the role of ferroptosis in the tumor microenvironment could reveal new strategies to target stromal cells and reprogram tumor-associated macrophages, improving the overall antitumor response (3). High-throughput screening and systems biology approaches are crucial for identifying novel ferroptosis-related biomarkers, which could aid in early diagnosis, prognosis, and personalized treatment planning. For example, biomarkers like GPX4 and ACSL4 have been recognized as key mediators of ferroptosis, and their clinical relevance warrants further validation (69, 70).

Finally, future studies should explore the heterogeneity of ferroptosis in different HCC subtypes to uncover tailored therapeutic strategies. Integrating multi-omics data, such as transcriptomics, metabolomics, and proteomics, could deepen our understanding of ferroptosis pathways and their interplay with other cell death mechanisms, paving the way for precision medicine approaches. By addressing these gaps, ferroptosis research has the potential to revolutionize HCC therapy and improve patient outcomes.

## 5 Highlights and limitations

Our study offers several unique contributions that distinguish it from other systematic reviews on ferroptosis and hepatocellular carcinoma (HCC). By employing advanced bibliometric methods, such as citation bursts, keyword co-occurrence analysis, and visualization tools like VOSviewer and CiteSpace, this study provides a quantitative and systematic evaluation of global research trends, collaborative networks, and emerging hotspots. Unlike traditional narrative reviews, it identifies novel research frontiers, including the intersection of ferroptosis with immunotherapy, tumor microenvironment, and biomarker discovery, which are not comprehensively discussed in existing reviews.

Additionally, the analysis of geographic and institutional collaboration patterns offers insights into the dynamics of international scientific cooperation, often overlooked in prior studies. This manuscript also provides actionable insights and expert opinions on integrating ferroptosis-targeted therapies into clinical applications, such as improving HCC prognosis and exploring synergistic effects with existing treatments. By highlighting interdisciplinary approaches like systems biology, high-throughput screening, and computational modeling, it underscores a forward-looking paradigm for ferroptosis research. These distinctive features position this study as a valuable resource for understanding the current state and future directions of ferroptosis in HCC.

Our study has several limitations that should be acknowledged. First, the data for this analysis was derived solely from the Web of Science Core Collection (WoSCC), which, while comprehensive, has recognized constraints. Previous studies have pointed out that WoSCC’s coverage is biased towards English-language publications and journals with high impact factors, potentially underrepresenting important findings published in non-English or lower-impact journals (33, 71). This selection bias could influence the generalizability of our results. Additionally, while WoSCC provides extensive metadata, limitations in the database’s structure might lead to challenges in accurately identifying certain entities, such as institutions with varying name formats or authors with similar names. Furthermore, the exclusion of other widely used databases like Scopus, which offers a broader coverage of regional and specialized studies, may have omitted relevant research, particularly in fields such as social sciences or regional innovations. The technical limitations of our bibliometric analysis tool have further contributed to the study’s constraints. For instance, categorizing publications into basic research, reviews, and clinical studies would provide valuable insights, but such classification requires manual inspection of each article. Given the large volume of publications analyzed in this study, this process is not feasible. We acknowledge the potential impact of this limitation on the depth of our analysis. The decision to use WoSCC was based on its robust indexing of multidisciplinary research, its strong emphasis on high-impact journals, and its advanced citation tracking tools, which are highly suitable for bibliometric analyses (72–75). However, Scopus is also a recognized authoritative database with distinct strengths, and its inclusion could provide complementary insights (76).

Future work could address these limitations by integrating data from multiple bibliometric databases, such as WoSCC and Scopus, and employing advanced algorithms and analytical tools to standardize and cross-reference metadata. This approach would enhance the representativeness and reliability of the research findings, ensuring a more comprehensive understanding of research trends.

## 6 Conclusion

This article provides a comprehensive overview of the research landscape on ferroptosis in hepatocellular carcinoma (HCC) through systematic bibliometric analysis and visualization techniques. The analysis indicates that China is at the forefront of publications related to ferroptosis and HCC, contributing the majority of research articles. The collaborative efforts among Chinese researchers and institutions, coupled with significant contributions from the United States and other countries, have cultivated a robust global research network.

The research hotspots in this field have gradually shifted from fundamental liver diseases and antioxidant mechanisms to cancer treatment targets and prognosis, reflecting the increasing importance of understanding disease outcomes in HCC research. Visualization of active and co-cited journals, authors, and references reveals the citation trajectory and knowledge flow within the field, pointing to the interdisciplinary and collaborative nature of ferroptosis research in HCC. The results of this analysis have laid a solid foundation for future research directions, offering valuable insights to clinicians and scientific investigators in the fields of oncology and cell death mechanisms.

In conclusion, the systematic analysis of the literature on ferroptosis in HCC has not only underscored the significant contributions from Chinese researchers but also highlighted the dynamic and collaborative nature of this research domain. The identified hotspots and trends provide a roadmap for future investigations, with the potential to advance our understanding of HCC pathogenesis and identify novel therapeutic strategies.

## Data Availability

The datasets presented in this study can be found in online repositories. The names of the repository/repositories and accession number(s) can be found below: https://clarivate.com.cn/solutions/web-of-science/.

## References

[1] Sung H Ferlay J Siegel RL Laversanne M Soerjomataram I Jemal A . Global cancer statistics 2020: GLOBOCAN estimates of incidence and mortality worldwide for 36 cancers in 185 countries. CA Cancer J Clin. (2021) 71:209–49. doi: 10.3322/caac.21660 33538338

[2] Wang W Wei C . Advances in the early diagnosis of hepatocellular carcinoma. Genes Dis. (2020) 7:308–19. doi: 10.1016/j.gendis.2020.01.014 PMC7452544 32884985

[3] He T Zou J Sun K Yang J . Global research status and frontiers on autophagy in hepatocellular carcinoma: a comprehensive bibliometric and visualized analysis. Int J Surg. (2024) 110:2788–802. doi: 10.1097/JS9.0000000000001343 PMC11093451 38376850

[4] Konyn P Ahmed A Kim D . Current epidemiology in hepatocellular carcinoma. Expert Rev Gastroenterol Hepatol. (2021) 15:1295–307. doi: 10.1080/17474124.2021.1991792 34624198

[5] Kao J-H Chen D-S . Changing disease burden of hepatocellular carcinoma in the Far East and Southeast Asia. Liver Int. (2005) 25:696–703. doi: 10.1111/j.1478-3231.2005.01139.x 15998418

[6] Gelli M Sebagh M Porcher R Romanelli E Vibert E Sa Cunha A . Liver resection for early hepatocellular carcinoma: preoperative predictors of non transplantable recurrence and implications for treatment allocation. Ann Surg. (2020) 272:820–6. doi: 10.1097/SLA.0000000000004259 32833755

[7] Jiang X Stockwell BR Conrad M . Ferroptosis: mechanisms, biology and role in disease. Nat Rev Mol Cell Biol. (2021) 22:266–82. doi: 10.1038/s41580-020-00324-8 PMC8142022 33495651

[8] Capelletti MM Manceau H Puy H Peoc'h K . Ferroptosis in liver diseases: an overview. Int J Mol Sci. (2020) 21. doi: 10.3390/ijms21144908 PMC7404091 32664576

[9] Ajoolabady A Tang D Kroemer G Ren J . Ferroptosis in hepatocellular carcinoma: mechanisms and targeted therapy. Br J Cancer. (2023) 128:190–205. doi: 10.1038/s41416-022-01998-x 36229582 PMC9902568

[10] Zhang D Man D Lu J Jiang Y Ding B Su R . Mitochondrial TSPO promotes hepatocellular carcinoma progression through ferroptosis inhibition and immune evasion. Adv Sci (Weinh). (2023) 10:e2206669. doi: 10.1002/advs.202206669 36994647 PMC10214260

[11] Lei G Zhuang L Gan B . The roles of ferroptosis in cancer: Tumor suppression, tumor microenvironment, and therapeutic interventions. Cancer Cell. (2024) 42:513–34. doi: 10.1016/j.ccell.2024.03.011 38593779

[12] Wang S Guo Q Zhou L Xia X . Ferroptosis: A double-edged sword. Cell Death Discovery. (2024) 10:265. doi: 10.1038/s41420-024-02037-9 38816377 PMC11139933

[13] Dang Q Sun Z Wang Y Wang L Liu Z Han X . Ferroptosis: a double-edged sword mediating immune tolerance of cancer. Cell Death Dis. (2022) 13:925. doi: 10.1038/s41419-022-05384-6 36335094 PMC9637147

[14] Yuan Y Zhai Y Chen J Xu X Wang H . Kaempferol ameliorates oxygen-glucose deprivation/reoxygenation-induced neuronal ferroptosis by activating nrf2/SLC7A11/GPX4 axis. Biomolecules. (2021) 11. doi: 10.3390/biom11070923 PMC8301948 34206421

[15] Zeng C Lin J Zhang K Ou H Shen K Liu Q . SHARPIN promotes cell proliferation of cholangiocarcinoma and inhibits ferroptosis via p53/SLC7A11/GPX4 signaling. Cancer Sci. (2022) 113:3766–75. doi: 10.1111/cas.v113.11 PMC9633309 35968603

[16] Yuan S Wei C Liu G Zhang L Li J Li L . Sorafenib attenuates liver fibrosis by triggering hepatic stellate cell ferroptosis via HIF-1α/SLC7A11 pathway. Cell Prolif. (2022) 55:e13158. doi: 10.1111/cpr.13158 34811833 PMC8780895

[17] Li Q Peng F Yan X Chen Y Zhou J Wu S . Inhibition of SLC7A11-GPX4 signal pathway is involved in aconitine-induced ferroptosis *in vivo* and *in vitro* . J Ethnopharmacol. (2023) 303:116029. doi: 10.1016/j.jep.2022.116029 36503029

[18] Yang R Gao W Wang Z Jian H Peng L Yu X . Polyphyllin I induced ferroptosis to suppress the progression of hepatocellular carcinoma through activation of the mitochondrial dysfunction via Nrf2/HO-1/GPX4 axis. Phytomedicine. (2024) 122:155135. doi: 10.1016/j.phymed.2023.155135 37856990

[19] Huang Q Li J Ma M Lv M Hu R Sun J . High−throughput screening identification of a small−molecule compound that induces ferroptosis and attenuates the invasion and migration of hepatocellular carcinoma cells by targeting the STAT3/GPX4 axis. Int J Oncol. (2023) 62. doi: 10.3892/ijo.2023.5490 PMC9946807 36825585

[20] Zhang J Luo Z Zheng Y Cai Q Jiang J Zhang H . A bibliometric study and visualization analysis of ferroptosis-inducing cancer therapy. Heliyon. (2023) 9:e19801. doi: 10.1016/j.heliyon.2023.e19801 37809417 PMC10559163

[21] Miao Y-D Quan W Dong X Gan J Ji C-F Wang J-T . A bibliometric analysis of ferroptosis, necroptosis, pyroptosis, and cuproptosis in cancer from 2012 to 2022. Cell Death Discovery. (2023) 9:129. doi: 10.1038/s41420-023-01421-1 37061535 PMC10105750

[22] Wu H Cheng K Li C . Ferroptosis, necroptosis, pyroptosis, and cuproptosis in cancer: a comparative bibliometric analysis. Cell Death Discovery. (2023) 9:238. doi: 10.1038/s41420-023-01542-7 37429861 PMC10333212

[23] Li G Liang Y Yang H Zhang W Xie T . The research landscape of ferroptosis in cancer: A bibliometric analysis. Front Cell Dev Biol. (2022) 10:841724. doi: 10.3389/fcell.2022.841724 35693942 PMC9174675

[24] Song L Zhang J Ma D Fan Y Lai R Tian W . A bibliometric and knowledge-map analysis of macrophage polarization in atherosclerosis from 2001 to 2021. Front Immunol. (2022) 13:910444. doi: 10.3389/fimmu.2022.910444 35795675 PMC9250973

[25] Wu P-N Liu J-L Fang M-J Fu X-S Wei J-L Wang Y . Global trends in colorectal cancer and metabolic syndrome research: a bibliometric and visualization analysis. Int J Surg. (2024) 110:3723–33. doi: 10.1097/JS9.0000000000001342 PMC11175816 38498393

[26] Kokol P . Discrepancies among Scopus and Web of Science, coverage of funding information in medical journal articles: a follow-up study. J Med Libr Assoc. (2023) 111:703–8. doi: 10.5195/jmla.2023.1513 PMC10361553 37483361

[27] van Eck NJ Waltman L . Software survey: VOSviewer, a computer program for bibliometric mapping. Scientometrics. (2010) 84:523–38. doi: 10.1007/s11192-009-0146-3 PMC2883932 20585380

[28] Arruda H Silva ER Lessa M Proença D Bartholo R . VOSviewer and bibliometrix. J Med Libr Assoc. (2022) 110:392–5. doi: 10.5195/jmla.2022.1434 PMC9782747 36589296

[29] Synnestvedt MB Chen C Holmes JH . CiteSpace II: visualization and knowledge discovery in bibliographic databases. AMIA Annu Symp Proc. (2005) 2005:724–8. PMC1560567 16779135

[30] Zhong D Li Y Huang Y Hong X Li J Jin R . Molecular mechanisms of exercise on cancer: A bibliometrics study and visualization analysis via citeSpace. Front Mol Biosci. (2021) 8:797902. doi: 10.3389/fmolb.2021.797902 35096970 PMC8794585

[31] Wu F Gao J Kang J Wang X Niu Q Liu J . Knowledge mapping of exosomes in autoimmune diseases: A bibliometric analysis (2002-2021). Front Immunol. (2022) 13:939433. doi: 10.3389/fimmu.2022.939433 35935932 PMC9353180

[32] Yang H-J Park C-K Chung M . Journal citation report 2020 and impact factor of journal of Korean neurosurgical society. J Korean Neurosurg Soc. (2021) 64:675–6. doi: 10.3340/jkns.2021.0208 PMC8435650 34503312

[33] Liu W Ni R Hu G . Web of Science Core Collection’s coverage expansion: the forgotten Arts & Humanities Citation Index? Scientometrics. (2024) 129:933–55. doi: 10.1007/s11192-023-04917-w

[34] Dixon SJ Lemberg KM Lamprecht MR Skouta R Zaitsev EM Gleason CE . Yang WS et al: Ferroptosis: an iron-dependent form of nonapoptotic cell death. Cell. (2012) 149:1060–72. doi: 10.1016/j.cell.2012.03.042 PMC3367386 22632970

[35] Sun X Ou Z Chen R Niu X Chen D Kang R . Activation of the p62-Keap1-NRF2 pathway protects against ferroptosis in hepatocellular carcinoma cells. Hepatology. (2016) 63:173–84. doi: 10.1002/hep.28251 PMC4688087 26403645

[36] Yang WS SriRamaratnam R Welsch ME Shimada K Skouta R Viswanathan VS . Regulation of ferroptotic cancer cell death by GPX4. Cell. (2014) 156:317–31. doi: 10.1016/j.cell.2013.12.010 PMC4076414 24439385

[37] Gao W Wang X Zhou Y Wang X Yu Y . Autophagy, ferroptosis, pyroptosis, and necroptosis in tumor immunotherapy. Signal Transduct Target Ther. (2022) 7:196. doi: 10.1038/s41392-022-01046-3 35725836 PMC9208265

[38] Liang C Zhang X Yang M Dong X . Recent progress in ferroptosis inducers for cancer therapy. Adv Mater. (2019) 31:e1904197. doi: 10.1002/adma.201904197 31595562

[39] Wang X Zhang H Guo Z Wang J Lu C Wang J . SNRPB promotes the progression of hepatocellular carcinoma via regulating cell cycle, oxidative stress, and ferroptosis. Aging (Albany NY). (2024) 16:348–66. doi: 10.18632/aging.205371 PMC10817389 38189879

[40] Liu D-L Wu M-Y Zhang T-N Wang C-G . Ferroptosis regulator modification patterns and tumor microenvironment immune infiltration characterization in hepatocellular carcinoma. Front Mol Biosci. (2022) 9:807502. doi: 10.3389/fmolb.2022.807502 35155577 PMC8832196

[41] Friedmann Angeli JP Krysko DV Conrad M . Ferroptosis at the crossroads of cancer-acquired drug resistance and immune evasion. Nat Rev Cancer. (2019) 19:405–14. doi: 10.1038/s41568-019-0149-1 31101865

[42] Wild CP Hall AJ . Primary prevention of hepatocellular carcinoma in developing countries. Mutat Res. (2000) 462:381–93. doi: 10.1016/S1383-5742(00)00027-2 10767647

[43] Ogasawara S Koroki K Kanzaki H Kobayashi K Kiyono S Nakamura M . Changes in therapeutic options for hepatocellular carcinoma in Asia. Liver Int. (2022) 42:2055–66. doi: 10.1111/liv.15101 34780081

[44] Wu C Li M Meng H Liu Y Niu W Zhou Y . Analysis of status and countermeasures of cancer incidence and mortality in China. Sci China Life Sci. (2019) 62:640–7. doi: 10.1007/s11427-018-9461-5 30900169

[45] Cao W Chen H-D Yu Y-W Li N Chen W-Q . Changing profiles of cancer burden worldwide and in China: a secondary analysis of the global cancer statistics 2020. Chin Med J (Engl). (2021) 134:783–91. doi: 10.1097/CM9.0000000000001474 PMC8104205 33734139

[46] Liu W Hu G Gu M . The probability of publishing in first-quartile journals. Scientometrics. (2015) 106:1273–6. doi: 10.1007/s11192-015-1821-1

[47] Xue Q Yan D Chen X Li X Kang R Klionsky DJ . Copper-dependent autophagic degradation of GPX4 drives ferroptosis. Autophagy. (2023) 19:1982–96. doi: 10.1080/15548627.2023.2165323 PMC10283421 36622894

[48] He F Zhang P Liu J Wang R Kaufman RJ Yaden BC . ATF4 suppresses hepatocarcinogenesis by inducing SLC7A11 (xCT) to block stress-related ferroptosis. J Hepatol. (2023) 79:362–77. doi: 10.1016/j.jhep.2023.03.016 PMC11332364 36996941

[49] Iseda N Itoh S Toshida K Tomiyama T Morinaga A Shimokawa M . Ferroptosis is induced by lenvatinib through fibroblast growth factor receptor-4 inhibition in hepatocellular carcinoma. Cancer Sci. (2022) 113:2272–87. doi: 10.1111/cas.v113.7 PMC9277415 35466502

[50] Ding Z Pan Y Shang T Jiang T Lin Y Yang C . URI alleviates tyrosine kinase inhibitors-induced ferroptosis by reprogramming lipid metabolism in p53 wild-type liver cancers. Nat Commun. (2023) 14:6269. doi: 10.1038/s41467-023-41852-z 37805657 PMC10560259

[51] Yang K Hu Y Qi H . Digital health literacy: bibliometric analysis. J Med Internet Res. (2022) 24:e35816. doi: 10.2196/35816 35793141 PMC9301558

[52] Gao Q Zhang G Zheng Y Yang Y Chen C Xia J . SLC27A5 deficiency activates NRF2/TXNRD1 pathway by increased lipid peroxidation in HCC. Cell Death Differ. (2020) 27:1086–104. doi: 10.1038/s41418-019-0399-1 PMC7206086 31367013

[53] Kudo Y Sugimoto M Arias E Kasashima H Cordes T Linares JF . PKCλ/ι Loss induces autophagy, oxidative phosphorylation, and NRF2 to promote liver cancer progression. Cancer Cell. (2020) 38. doi: 10.1016/j.ccell.2020.05.018 PMC7423690 32589943

[54] Qian H Chao X Williams J Li T Yang L Ding W-X . Autophagy in liver diseases: A review. Mol Aspects Med. (2021) 82:100973. doi: 10.1016/j.mam.2021.100973 34120768 PMC9585624

[55] Wang L Hu T Shen Z Zheng Y Geng Q Li L . Inhibition of USP1 activates ER stress through Ubi-protein aggregation to induce autophagy and apoptosis in HCC. Cell Death Dis. (2022) 13:951. doi: 10.1038/s41419-022-05341-3 36357365 PMC9649627

[56] Oura K Morishita A Tani J Masaki T . Tumor immune microenvironment and immunosuppressive therapy in hepatocellular carcinoma: A review. Int J Mol Sci. (2021) 22. doi: 10.3390/ijms22115801 PMC8198390 34071550

[57] Sangro B Sarobe P Hervás-Stubbs S Melero I . Advances in immunotherapy for hepatocellular carcinoma. Nat Rev Gastroenterol Hepatol. (2021) 18:525–43. doi: 10.1038/s41575-021-00438-0 PMC8042636 33850328

[58] Foerster F Gairing SJ Ilyas SI Galle PR . Emerging immunotherapy for HCC: A guide for hepatologists. Hepatology. (2022) 75:1604–26. doi: 10.1002/hep.32447 PMC9117522 35253934

[59] Bicer F Kure C Ozluk AA El-Rayes BF Akce M . Advances in immunotherapy for hepatocellular carcinoma (HCC). Curr Oncol. (2023) 30:9789–812. doi: 10.3390/curroncol30110711 PMC10670350 37999131

[60] Lee Y-T Fujiwara N Yang JD Hoshida Y . Risk stratification and early detection biomarkers for precision HCC screening. Hepatology. (2023) 78:319–62. doi: 10.1002/hep.32779 PMC9995677 36082510

[61] Yu SJ . Immunotherapy for hepatocellular carcinoma: Recent advances and future targets. Pharmacol Ther. (2023) 244:108387. doi: 10.1016/j.pharmthera.2023.108387 36948423

[62] Yao F Deng Y Zhao Y Mei Y Zhang Y Liu X . A targetable LIFR-NF-κB-LCN2 axis controls liver tumorigenesis and vulnerability to ferroptosis. Nat Commun. (2021) 12:7333. doi: 10.1038/s41467-021-27452-9 34921145 PMC8683481

[63] Johnson P Zhou Q Dao DY Lo YMD . Circulating biomarkers in the diagnosis and management of hepatocellular carcinoma. Nat Rev Gastroenterol Hepatol. (2022) 19:670–81. doi: 10.1038/s41575-022-00620-y 35676420

[64] Yang F Hilakivi-Clarke L Shaha A Wang Y Wang X Deng Y . Metabolic reprogramming and its clinical implication for liver cancer. Hepatology. (2023) 78:1602–24. doi: 10.1097/HEP.0000000000000005 PMC10315435 36626639

[65] Hong J Zheng W Cai X . Small-molecule high-throughput screening identifies an MEK inhibitor PD198306 that enhances sorafenib efficacy via MCL-1 and BIM in hepatocellular carcinoma cells. Comb Chem High Throughput Screen. (2023) 26:1364–74. doi: 10.2174/1386207325666220830145026 PMC9971357 36043792

[66] Calderaro J Seraphin TP Luedde T Simon TG . Artificial intelligence for the prevention and clinical management of hepatocellular carcinoma. J Hepatol. (2022) 76:1348–61. doi: 10.1016/j.jhep.2022.01.014 PMC9126418 35589255

[67] Walakira A Skubic C Nadižar N Rozman D Režen T Mraz M . Integrative computational modeling to unravel novel potential biomarkers in hepatocellular carcinoma. Comput Biol Med. (2023) 159:106957. doi: 10.1016/j.compbiomed.2023.106957 37116239

[68] Wang W Green M Choi JE Gijón M Kennedy PD Johnson JK . CD8+ T cells regulate tumor ferroptosis during cancer immunotherapy. Nature. (2019) 569:270–4. doi: 10.1038/s41586-019-1170-y PMC6533917 31043744

[69] Yang WS Stockwell BR . Ferroptosis: death by lipid peroxidation. Trends Cell Biol. (2016) 26:165–76. doi: 10.1016/j.tcb.2015.10.014 PMC4764384 26653790

[70] Doll S Proneth B Tyurina YY Panzilius E Kobayashi S Ingold I . ACSL4 dictates ferroptosis sensitivity by shaping cellular lipid composition. Nat Chem Biol. (2017) 13:91–8. doi: 10.1038/nchembio.2239 PMC5610546 27842070

[71] Martín-Martín A Orduna-Malea E Delgado López-Cózar E . Coverage of highly-cited documents in Google Scholar, Web of Science, and Scopus: a multidisciplinary comparison. Scientometrics. (2018) 116:2175–88. doi: 10.1007/s11192-018-2820-9 PMC7505221 32981987

[72] Falagas ME Pitsouni EI Malietzis GA Pappas G . Comparison of PubMed, Scopus, Web of Science, and Google Scholar: strengths and weaknesses. FASEB J. (2008) 22:338–42. doi: 10.1096/fj.07-9492LSF 17884971

[73] Brandt JS Hadaya O Schuster M Rosen T Sauer MV Ananth CV . A bibliometric analysis of top-cited journal articles in obstetrics and gynecology. JAMA Netw Open. (2019) 2:e1918007. doi: 10.1001/jamanetworkopen.2019.18007 31860106 PMC6991228

[74] Giménez-Espert MDC Prado-Gascó VJ . Bibliometric analysis of six nursing journals from the Web of Science, 2012-2017. J Adv Nurs. (2019) 75:543–54. doi: 10.1111/jan.13868 30289557

[75] Aski SK Akbari R Hantoushzadeh S Ghotbizadeh F . A bibliometric analysis of Intrauterine Growth Restriction research. Placenta. (2020) 95:106–20. doi: 10.1016/j.placenta.2020.03.010 32452397

[76] Rew D . SCOPUS: Another step towards seamless integration of the world's medical literature. Eur J Surg Oncol. (2010) 36:2–3. doi: 10.1016/j.ejso.2009.08.001 19716258

